# Fabrication of Subnanometer-Precision Nanopores in Hexagonal Boron Nitride

**DOI:** 10.1038/s41598-017-12684-x

**Published:** 2017-11-08

**Authors:** S. Matt Gilbert, Gabriel Dunn, Amin Azizi, Thang Pham, Brian Shevitski, Edgar Dimitrov, Stanley Liu, Shaul Aloni, Alex Zettl

**Affiliations:** 1 Department of Physics, University of California at Berkeley, Berkeley, CA 94720 USA; 2 Materials Sciences Division, Lawrence Berkeley National Laboratory, Berkeley, CA 94720 USA; 3 Kavli Energy NanoScience Institute at the University of California, Berkeley and the Lawrence Berkeley National Laboratory, Berkeley, CA 94720 USA; 4 Molecular Foundry, Lawrence Berkeley National Laboratory, Berkeley, CA 94720 USA

## Abstract

We demonstrate the fabrication of individual nanopores in hexagonal boron nitride (h-BN) with atomically precise control of the pore shape and size. Previous methods of pore production in other 2D materials typically create pores with irregular geometry and imprecise diameters. In contrast, other studies have shown that with careful control of electron irradiation, defects in h-BN grow with pristine zig-zag edges at quantized triangular sizes, but they have failed to demonstrate production and control of isolated defects. In this work, we combine these techniques to yield a method in which we can create individual size-quantized triangular nanopores through an h-BN sheet. The pores are created using the electron beam of a conventional transmission electron microscope; which can strip away multiple layers of h-BN exposing single-layer regions, introduce single vacancies, and preferentially grow vacancies only in the single-layer region. We further demonstrate how the geometry of these pores can be altered beyond triangular by changing beam conditions. Precisely size- and geometry-tuned nanopores could find application in molecular sensing, DNA sequencing, water desalination, and molecular separation.

## Introduction

Nanoporous materials have received significant attention due to intriguing basic science issues^1^ and a range of applications: molecular sensing^2^, DNA sequencing^3–5^, water desalination^6^, and molecular separation^7,8^ to name a few. Nanopores in 2D materials such as graphene^9–11^, hexagonal boron nitride (h-BN)^12,13^, molybdenum disulfide (MoS_2_)^14^, and tungsten disulfide (WS_2_)^15^ have gained particular attention, as limiting the pore’s channel length to a single or few atoms makes for more sensitive sensors^16^ or more energy efficient filters^17,18^. Additionally, the crystalline nature of layered materials creates pores in which the chemical groups at the pore’s edge differ from those of the rest of the sheet, allowing for chemical functionalization to improve performance^19^.

The performance in each of the applications mentioned above relies critically on precise control of pore size. In molecular sensing, a tighter pore size results in a larger signal as a target molecule more fully blocks the channel, leading to improved sensing accuracy^20,21^. DNA sequencing benefits from this improved signal, particularly for nanopores with diameters ranging from 1–3 nm, but also these smaller and more precise pore sizes help slow the passage of DNA through the pore – solving a common problem facing many solid state nanopore devices aimed at sequencing^22,23^. Atomically precise pore sizes would allow for molecular sieves and gas separation systems capable of isolating smaller molecules and separating species with atomic scale differences in size and shape^8^. While other techniques have had success in creating precise nanopores in 2D materials^20,24–27^, they have lacked the subnanometer precision and pore sizes necessary to realize their full potential in a range of applications.

In this work, we describe and establish a procedure for the fabrication of individual nanopores in few-layer h-BN with atomically precise control of pore size from few-atom vacancies to several nanometer side-length through careful control of transmission electron microscope (TEM) electron beam conditions. It has been previously demonstrated that when h-BN is exposed under 80 kV electron irradiation in TEM that regular atomically precise triangle defects form^28–33^. Due to the preferential ejection of boron, attributed either to electron knock-on effects or selective chemical etching by atomic species present in the TEM, metastable nitrogen terminated zig-zag edges form and preserve a triangular shape under the electron beam. As these undercoordinated nitrogen atoms are ejected a new nitrogen zig-zag edge is exposed allowing for the precise quantized growth of the triangle defect^32,34^. In all of the previous work, however, the defects studied, despite growing at precise increments, were not controlled in number or size; many defects were formed and the rate of growth was left uncontrolled. In other studies, in which the number and position of the defects in h-BN were controlled for nanopore studies, the defects formed were irregular in shape and larger than 5 nm due to the use of high currents or voltages in order to readily create single pores^12,13^.

**Figure 1:**
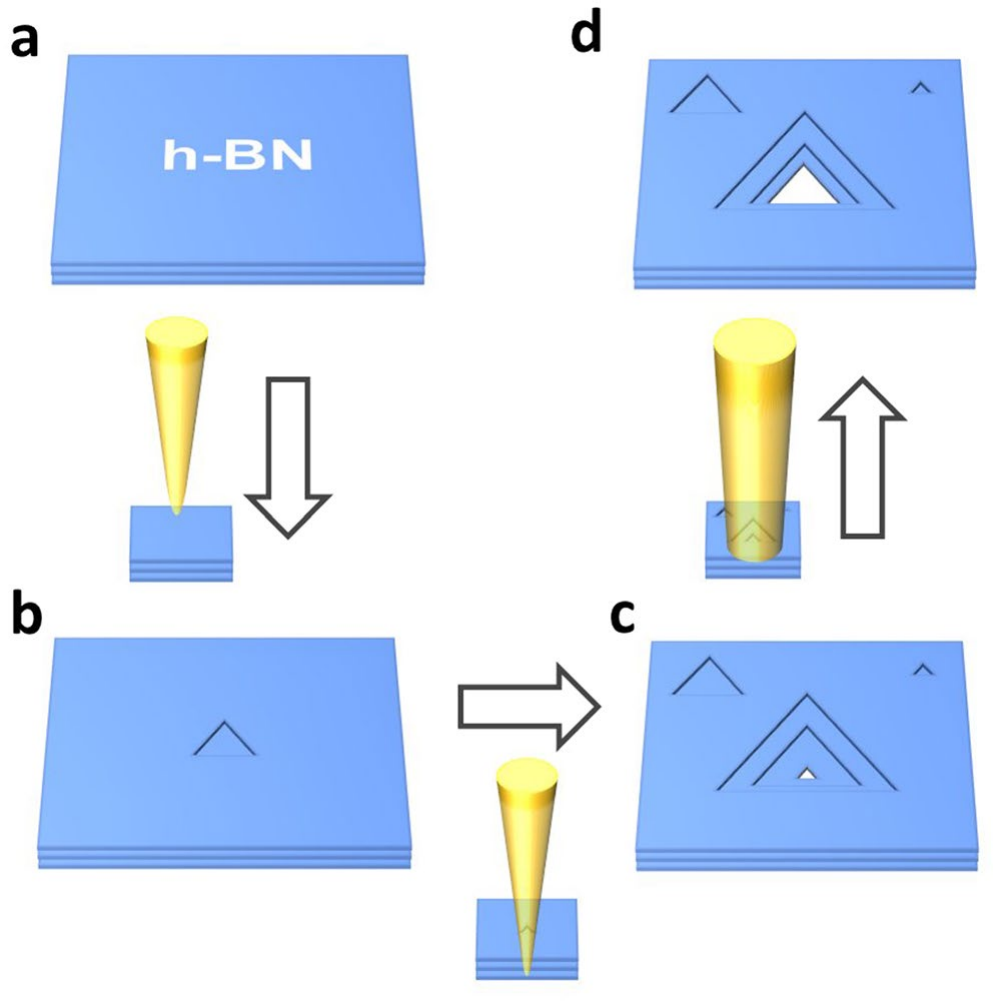
Schematic of our nanopore fabrication method. (**a**) Starting from pristine h-BN a TEM electron beam is condensed to 10–20 nm area. (**b**) Triangular defects form under the condensed beam, mostly near the center, allowing for the stripping of the h-BN layer by layer. (**c**) After the formation of a single few-atom vacancy in the final layer, the beam is spread. (**d**) Under a lower beam energy density, the pore is grown to the desired size.

Here, we develop a method that combines the strengths of these approaches by allowing for both the nucleation of single nanopores and for the precise growth of these defects in h-BN from few atom vacancies to several nanometer side-lengths. This process can be accomplished in a conventional TEM by only modifying the beam conditions and does not need an advanced aberration-corrected TEM. Our process for creating an individual nanopore is shown schematically in Fig. 1, and consists of two main steps. First, with a high current density focused electron beam, we strip away layers of h-BN over a small area until we are left with a single few-atom vacancy in a small single-layer region. Second, the electron beam is spread and the preferential growth of the nanopore under a diffuse electron beam is monitored, while the formation of additional vacancies and pores is suppressed.

## Results and Discussion

**Figure 2:**
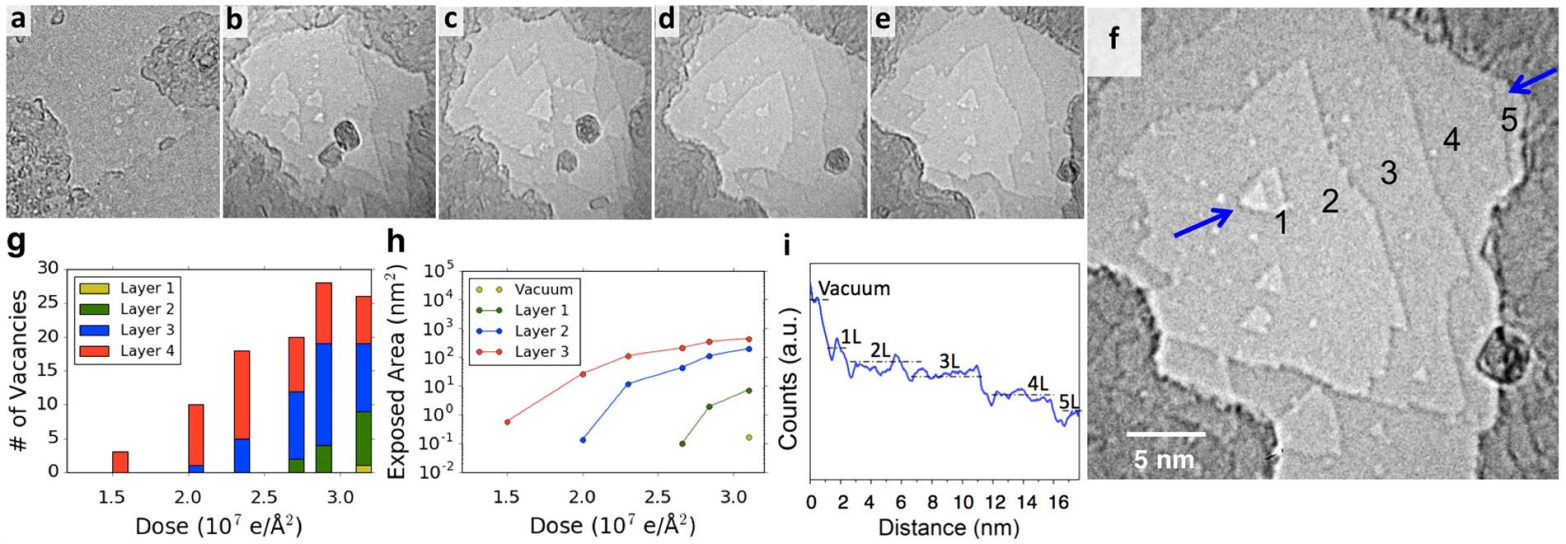
Layer by layer stripping of multilayer h-BN. (**a**–**f**) TEM time series showing the formation of defects and stripping of layers at doses of (**a**) 0, (**b**) 2.0 × 10^7^ e/Å^2^, (**c**) 2.3 × 10^7^ e/Å^2^ (11 min), (**d**) 2.8 × 10^7^ e/Å^2^ (15 min), and (**e**) 3.1 × 10^7^ e/Å^2^ (18.5 min) under a 10–20 nm condensed electron beam with a current density of 37 A/cm^2^. After frame (**e**) the beam is expanded with a current density of 3 A/cm^2^ to produce the single nanopore shown in (**f**). In (**f**) the layer numbers are denoted. (**g**) The number of continuous vacancies present in each layer of the sample as a function of dose. (**h**) A graph depicting the amount of area exposed of a given layer or below, note that the area exposed of each layer or below goes asymptotically towards probe size. (**i**) Grayscale count profile along the path between the two arrows in (**f**). The difference between layer 1 and vacuum is larger than between other layers. The size in nanometers of frames (**a**–**f**) are constant.

In order to prepare individual nanopores in multilayer h-BN, we first strip away layers in a localized region by milling with a condensed electron beam in a JEOL 2010 TEM operated at 80 kV. As shown in Fig. 2(a–f), by using a beam condensed to a diameter of 10–20 nm at a current density of 37 A/cm^2^, vacancies are readily formed in each layer sequentially and steadily grow with dose (Fig. 2(g) and (h)), effectively stripping away layer by layer. Under these beam conditions, this process proceeds fairly slowly giving a good deal of control; Fig. 2(h) shows the area of the sample exposed for a given layer or below. In this experiment, a dose of approximately 6 × 10^6^ e/Å^2^ or 4.5 minutes is required to strip to each successive layer under the condensed beam. Vacancies in the final single-layer region of the h-BN are identified by a larger contrast difference (Fig. 2(i)), allowing us to stop the milling after an isolated vacancy has been introduced in the single-layer region.

**Figure 3:**
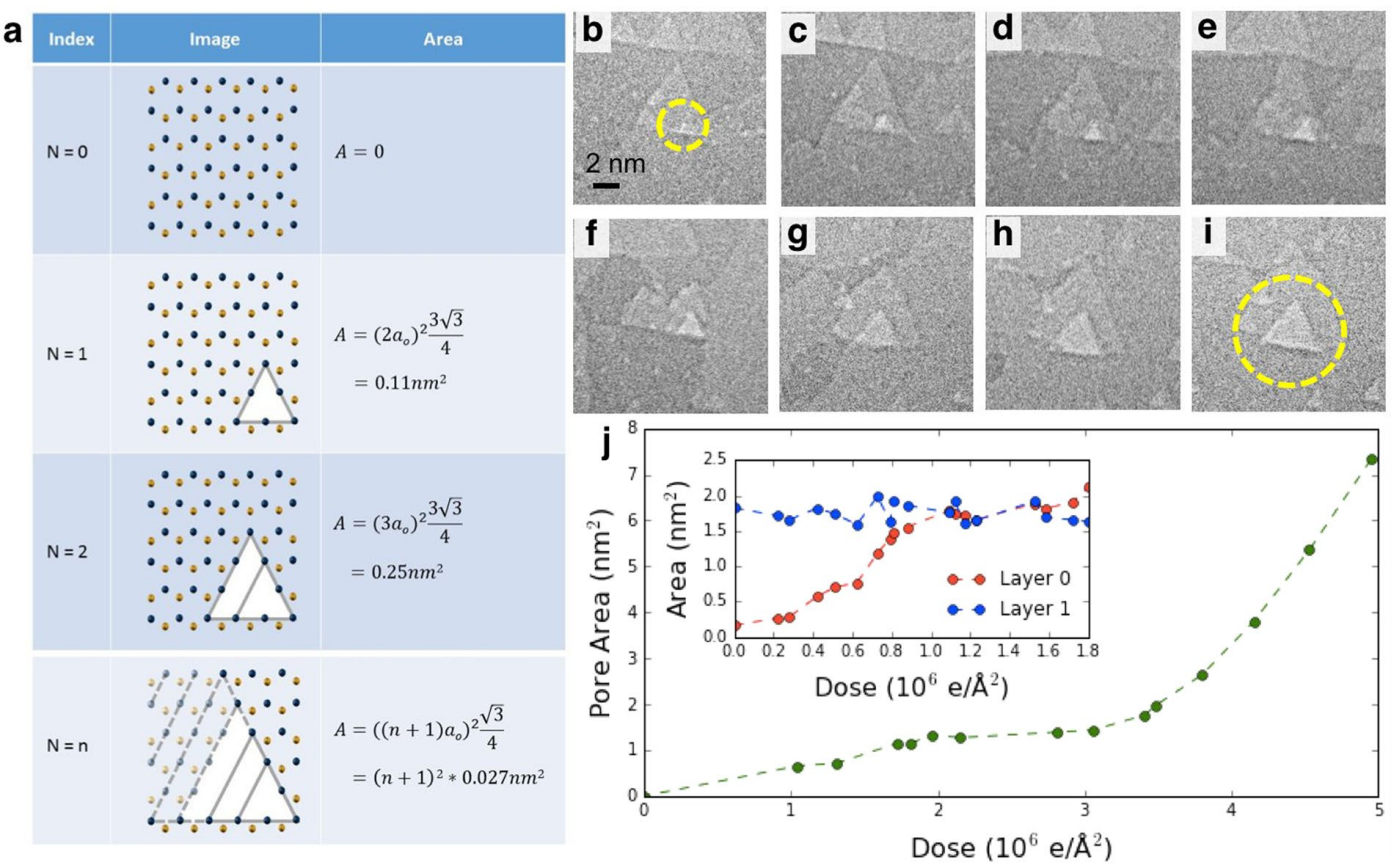
Metastable quantized growth of triangular nanopores. (**a**) For each quantized triangular pore size, an image shows the atomic configuration of the pore and the resultant pore area. Nitrogen and boron atoms are depicted in blue and gold respectively. The spacing between neighboring boron and nitrogen atoms, a_o_, is 1.45 Å. (**b**–**i**) A time series showing the quantized growth of a triangular nanopore in h-BN from a few-atom vacancy to approximately 8 nm^2^ under a beam current of 6 A/cm^2^. (**b**) mono to few-atom vacancy formed in bottom h-BN sheet, circled in yellow. (**c**–**i**) Metastable quantized growth of nanopore. Shuttering the electron beam irradiation causes the pore growth to cease. Images are taken at roughly 2 minute intervals. (**j**) A plot of pore area versus dose for the images shown in (**a**–**h**). The inset shows a similar growth of pore area versus dose for the nanopore shown in Fig. 2(f) and a neighboring vacancy of similar size in the second layer under a beam current of 3 A/cm^2^. Unlike the pore that spans the full thickness of the h-BN, the vacancy that sits on top of another layer of h-BN does not grow at an appreciable rate.

After a small vacancy has been produced, a lower energy density beam allows us to grow the nanopore in quantized triangles of precise size, while new vacancy formation is inhibited. As the nanopore grows, it favors a triangular geometry and is metastable at each quantized triangle size, allowing for easily reproducible, highly regular pore geometry, as shown in Fig. 3(a). Figure 3(b–i) show an example of the growth of a single nanopore from a few-atom vacancy to 8 nm^2^ with the beam current density reduced to 6 A/cm^2^, where the pore growth as a function of dose can be seen in Fig. 3(j). As has been described previously, electron knock-on effects and/or selective chemical etching due to gases present in the TEM column preferentially eject boron atoms and preserves nitrogen zig-zag edge termination^28–32,35^. Hence, each of the triangular pores created is reliably nitrogen terminated, desirable for many nanopore applications where controlling end-group chemistry is critical. When the nanopore has reached its desired size, the beam can be fully expanded or blanked to cease pore growth.

By further lowering the beam current density, the nanopore in the single-layer region can be preferentially grown while similar defects in the multilayer region remain static. This is possible because vacancies in multi-layer regions appear to be stabilized, with inhibited further evolution, by the supporting h-BN layers. This is shown in the inset in Fig. 3(j), where over the course of exposing the sample with a total electron dose of 1.8 × 10^6^ e/Å^2^ over 16 minutes at beam current of 3 A/cm^2^, the pore that spans the bottom layer grows by 10-fold while the similar vacancy in the second layer (shown in Fig. S1) remains constant in size.

**Figure 4:**
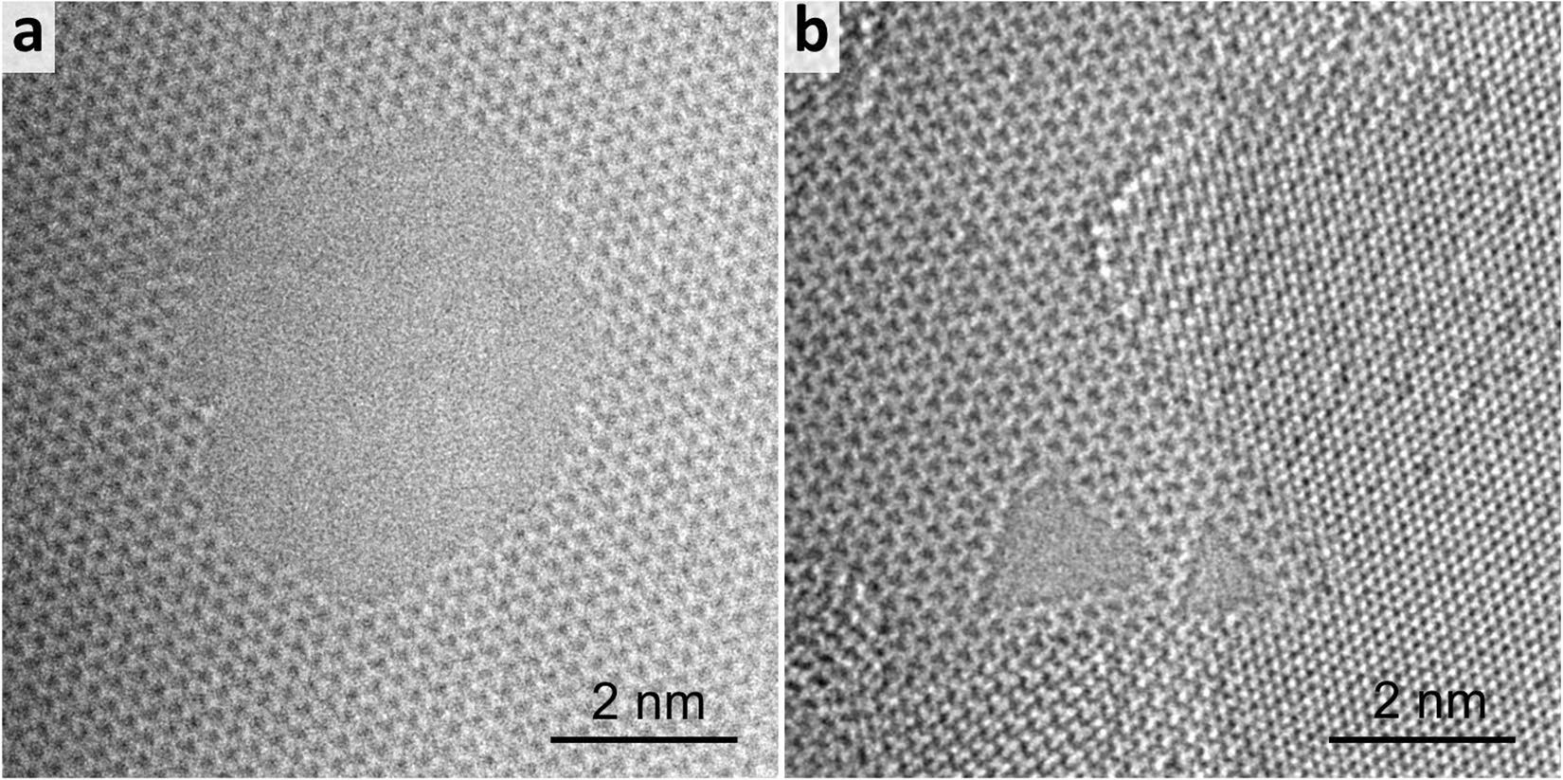
Nanopores produced in graphene (left) and h-BN(right) *in-situ* under diffuse electron irradiation using TEM mode on the National Center for Electron Microscopy’s TEAM 0.5 aberration corrected microscope at 80 kV. The graphene nanopore has irregular edges with no preferred termination whereas the h-BN pore has pristine zig-zag edges.

While this method of vacancy seeding and pore growth may be extended to other 2D materials, h-BN nanopores prepared in this manner hold key advantages. When a similar method is used on graphene, the pore geometry is highly irregular, as demonstrated in Fig. 4 and previous work^20,25,36^. When irradiating a graphene sample under a diffuse electron beam using HRTEM, the edges of defects (Fig. 4a) do not pick any specific orientation as compared to h-BN (Fig. 4b) which has atomically pristine zig-zag edges. These zig-zag edges can confer an additional stability to the material versus graphene which can suffer from the uncontrolled addition of functional groups on its irregular edges after exposure to air^37,38^. Initiating pore growth is also significantly more difficult for graphene, requiring a much higher energy or beam current and a fully condensed beam, which yields larger starting vacancies.

Due to the diatomic basis of the two-dimensional transition metal dichalcogenides (TMDs) such as MoS_2_ or WSe_2_, they may also be possible candidates for similar triangular nanopores. However, Komsa *et al*.^39^ have reported that under electron irradiation the formation and aggregation of chalcogenide vacancies occurs preferentially over metal ejection. Feng *et al*.^14^ report the formation of nanopores in MoS_2_ by irradiating with a condensed electron beam, but these nanopores do not appear to take on any special geometry.

For the applications requiring the nanopore to serve as a fluidic channel, such as DNA sequencing, h-BN has been shown to have additional advantages over other 2D materials, for example a h-BN nanopore is more hydrophilic than a graphene nanopore due to the BN polar bond structure^13^. This allows for improved wetting for h-BN membranes, and consequent ionic conduction and DNA translocation through h-BN nanopores^12,13^. The insulating nature of h-BN may also be an advantage over graphene and TMDs for reducing noise in transmembrane current measurements and in instances where integrating patterned electronics on the substrate is necessary such as in nanoribbon and nanowire or plasmonic nanoantenna detection of nanopores^40–42^.

While we demonstrate that high precision nanopores can be created using conventional TEM, the TEM irradiation process may be difficult to scale for applications requiring more than one nanopore. However, chemical routes such as hydrogen annealing have demonstrated the ability to form triangle vacancies in h-BN^43^. By optimizing such a chemical process or by applying local heating as Nam *et al*.^40^, number and size of defects could be controlled outside of the TEM at much larger throughput. Other groups have also shown that defects in h-BN can be produced at lower accelerating voltages using a scanning electron microscope (SEM)^44^; further studies of this process might allow standard electron beam lithography exposures to create precise nanopores in h-BN.

The nitrogen terminated triangular defect geometry described above is not the only stable geometry possible for h-BN under electron irradiation; other pore geometries and end groups can be formed under different conditions^30,35,45,46^. In particular, previous work has shown that by elevating the temperature of the h-BN while it is exposed that hexagonal defects can be formed^35,45^. The formation of these hexagonal defects is justified by the revelation that there is an energy barrier between the hexagonal and triangular states which the heat provides the energy to overcome in these prior works.

**Figure 5:**
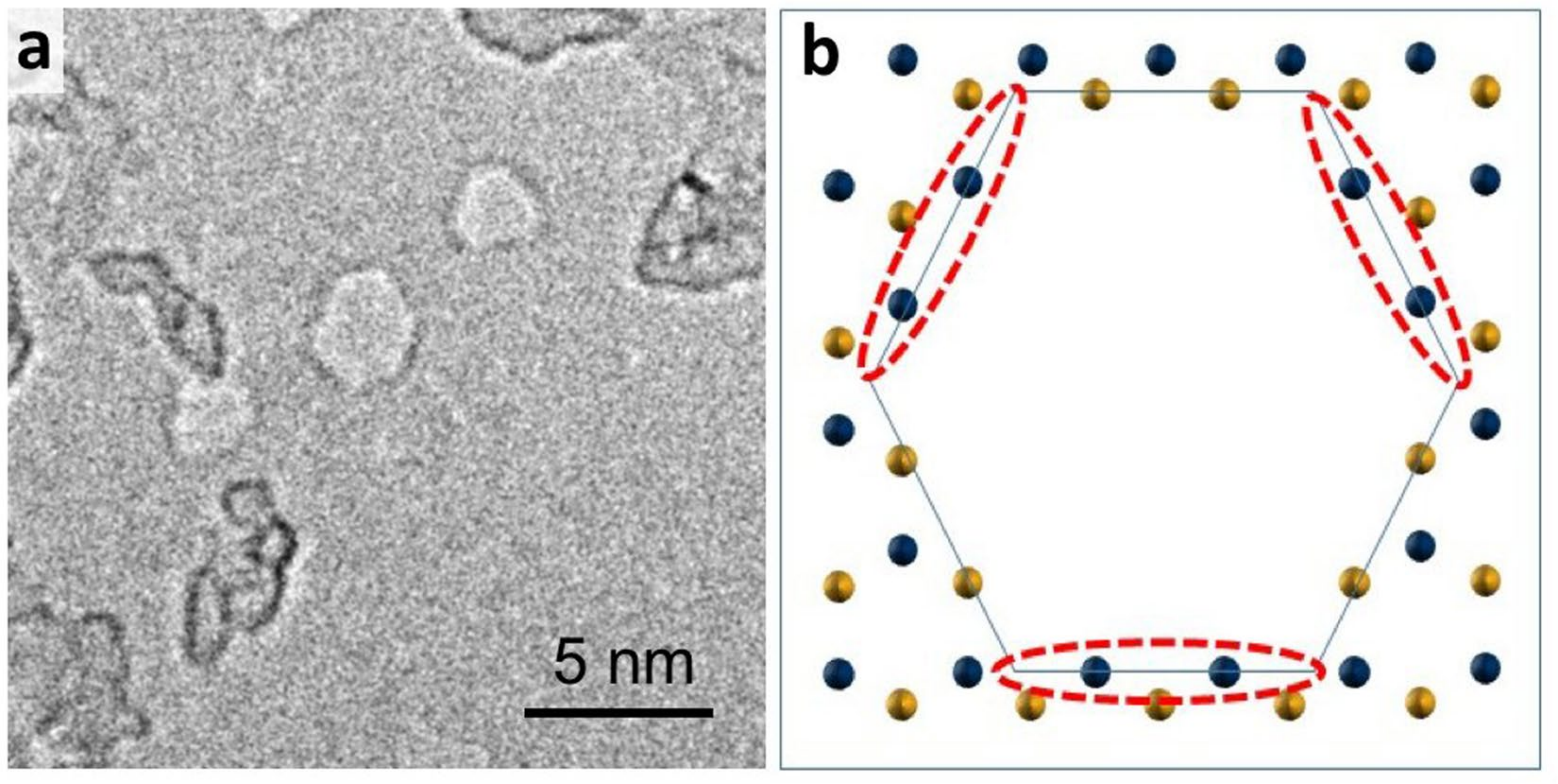
(**a**) Hexagonal h-BN pore created by condensing the beam at higher spot sizes to achieve higher current density. (**b**) Alternating boron and nitrogen facets as demonstrated in ref.^45^. Boron and Nitrogen represented in gold and blue respectively. The nitrogen terminated facets that normally form the edges of a triangular nanopore are circled in red and are stable at low beam currents while the boron terminated edges are not.

Here we show that by increasing the beam current density we are also able to form hexagonal defects using a conventional TEM. By using a smaller spot size to increase the condensed beam current to approximately 70 A/cm^2^, hexagonal defects are formed, as shown in Fig. 5. As has been described elsewhere, in order to create hexagonal defects, both boron and nitrogen edges must be present^45^. These hexagons likely form because the extra beam current compensates for the energy difference between boron and nitrogen zig-zag edge similar to how heating has provided this extra energy in the prior literature^35,45^. The hexagonal pores can be steadily increased in size if the beam current remains around 70 A/cm^2^; however, if the beam current is then reduced to a current favorable for triangular nanopore growth, the hexagonal pore will grow towards a triangular shape as shown in Figure S4.

In summary, we have developed a method for fabricating individual h-BN nanopores with pore sizes that can be controlled with atomic precision. By careful control of beam conditions, electron beam irradiation can be used to strip away h-BN layers and create vacancies and can be used for nanopore growth. Under the correct beam conditions, this pore growth is limited to pores in single-layer h-BN and is inhibited in multi-layer regions. The pore geometry and end-group chemistries are both highly regular and controllable. Furthermore, we demonstrate in this work that these atomically precise nanopores can be created using a conventional TEM. Using an aberration corrected TEM allows refined characterization. h-BN nanopores with tunable pore size and geometry can find application in DNA sequencing (*e*.*g*. the pores < 2 nm have approximately the same dimensions as single-strand DNA) and molecular sensing^20,21^ where smaller pores and more precise end-group functionalization could increase sensitivity and performance respectively, in water desalination where better tailored pore sizes and functionalization could improve performance and efficiency^47^, and in molecular separation, where precise pore size and end group control would allow for better discrimination between like chemical species^8^.

## Methods

The h-BN for this work is prepared by chemical vapor deposition on copper. Prior to growth, a 1 cm × 4 cm copper foil (Sigma Aldrich) is placed in acetic acid for 10 min followed by rinsing in 3 DI water baths. The sample is then annealed at 1020 °C under a mixture of 100 sccm H_2_ and 300 sccm Ar environment for 2 hours. After annealing, the argon is shut off and 100 mg of ammonia-borane (Sigma Aldrich 97%) precursor is heated in a connected upstream one-end sealed quartz tube to 70–90 °C for 30 minutes. The precursor is then cooled quickly while the copper foil is allowed to cool slowly under 10 sccm H2. This process yields 3–5 layer h-BN.

The h-BN is then either transferred to SiN membranes containing a single 100 nm hole via a PMMA assisted transfer^48^ or to holey carbon TEM grids (Ted Pella) as in ref.^49^. For the polymer assisted transfer, PMMA A4 is spun onto the top side of the copper foil. The foil is then floated on a bath of sodium persulfate in order to etch away the underlying copper, leaving the h-BN/PMMA film floating at the surface. A commercial TEM window chip (Norcada) with a 500 × 500 µm, 50 nm thick membrane is prepared for nanopore suspension by creating a 20–100 nm pore in the suspended nitride membrane. The hole is either formed using a Helium Ion Beam at 25 kV or by electron beam milling using an 80 kV condensed beam for 30–60 min. The modified TEM grid is used to scoop up the h-BN/PMMA film to transfer it over the nitride membrane pore. The PMMA is removed by annealing the chip at 350 °C in an evacuated tube furnace under 300 sccm argon, 100 sccm hydrogen.

Individual nanopores in h-BN are prepared using a JEOL 2010 TEM operated at 80 kV as described in the text above. The electron beam is condensed to a 10–20 nm diameter at spot size 3, alpha = 3 with a beam current of 37 A/cm^2^. The beam is expanded periodically to check the progress of this step. Based on the data presented in Fig. 2, one pore forms in a newly exposed layer roughly every 5 minutes. It is therefore straightforward to monitor and observe the formation of new defects by frequently spreading the beam and acquiring images. After the formation of the first vacancy between the first layer and vacuum, as identified by the difference in contrast and the accelerated growth rate of the vacancy, the beam is left expanded at a beam current 3 A/cm^2^ at spot size 3 or 6 A/cm^2^ at spot size 1. The size and number of vacancies as described in Figs 2(g) and 3(j) are extracted in post-analysis based on the recorded images taken during the pore formation.

### Data Availability

The datasets generated during the current study are available from the corresponding author on reasonable request.

## Electronic supplementary material

Supplementary Figures
