# Supplementary material for: Fabrication of Subnanometer-Precision Nanopores in Hexagonal Boron Nitride
Source: Sci Rep. 2017 Nov 8;7:15096. doi: 10.1038/s41598-017-12684-x (PMC5678191; doi:10.1038/s41598-017-12684-x)
Supplement: Supplementary file 1 — Supplementary Figures [file 41598_2017_12684_MOESM1_ESM.pdf]

Supplementary Figures for

Fabrication of Subnanometer-Precision Nanopores in Hexagonal Boron Nitride

S. Matt Gilbert[‡,1,2,3], Gabriel Dunn[‡,1,2,3], Amin Azizi[1,3], Thang Pham[1,2,3], Brian Shevitski[1,2,3,4], Edgar Dimitrov[1,3], Stanley Liu[1,3], Shaul Aloni[4] and Alex Zettl*[,1,2,3].

[1] Department of Physics, University of California at Berkeley, Berkeley, CA 94720, USA
[2] Materials Sciences Division, Lawrence Berkeley National Laboratory, Berkeley, CA 94720, USA
[3] Kavli Energy NanoScience Institute at the University of California, Berkeley and the Lawrence Berkeley National Laboratory, Berkeley, CA 94720, USA
[4] Molecular Foundry, Lawrence Berkeley National Laboratory, Berkeley, CA 94720, USA
[‡] SMG and GD contributed equally to this work.

Correspondence and requests for materials should be addressed to A.Z. (email azettl@berkeley.edu)

Figure S1

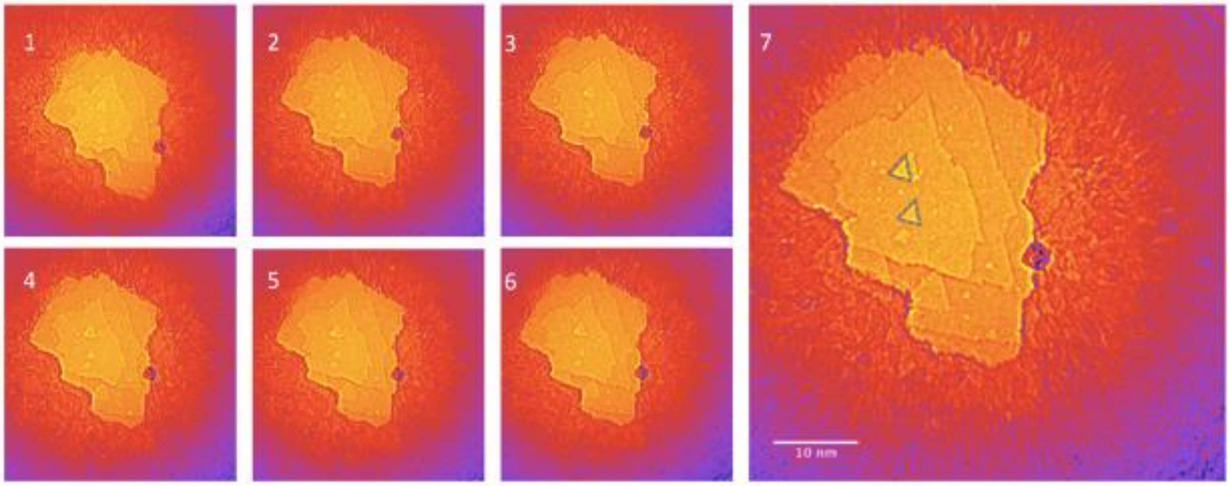

A time series showing the growth of the nanopore the nanopore highlighted in figure 2(f) from a few-atom vacancy to 1.9 nm side length. In figure 3 (j) (inset) the growth of the nanopore highlighted in panel 7 on the left is compared to the growth of a vacancy that is not backed to vacuum highlighted on the right.

Figure S2

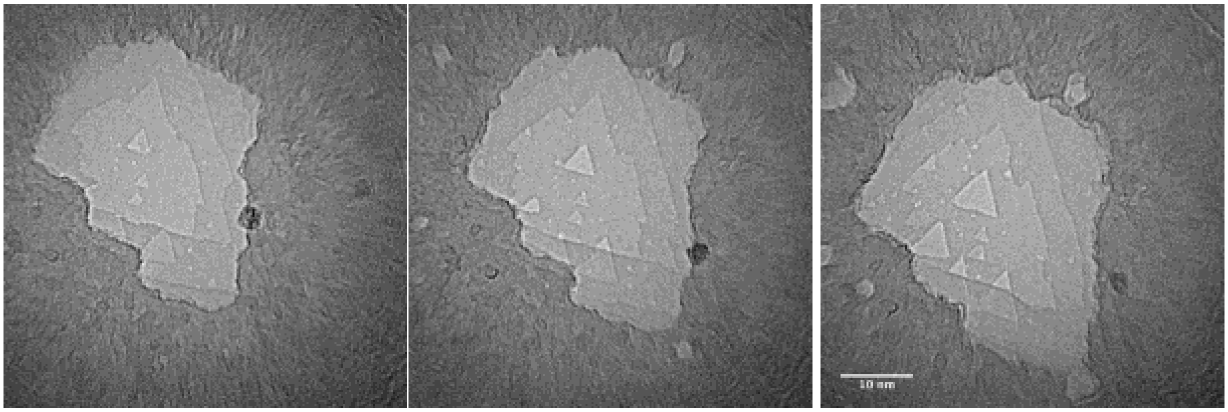

A time series of the nanopore formed in figure 2 showing the growth of the nanopore from 2 nm to 7 nm. In the middle panel, two other nanopores have formed. In the right panel four additional pores have formed.

Figure S3

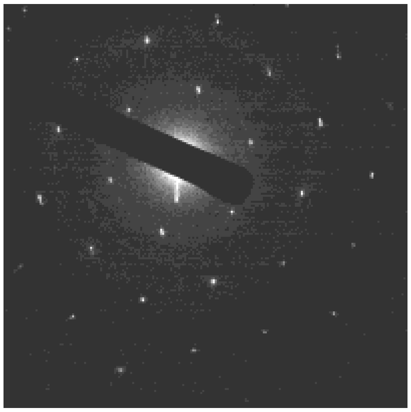

Selected area electron diffraction of the sample studied for these measurements.

Figure S4

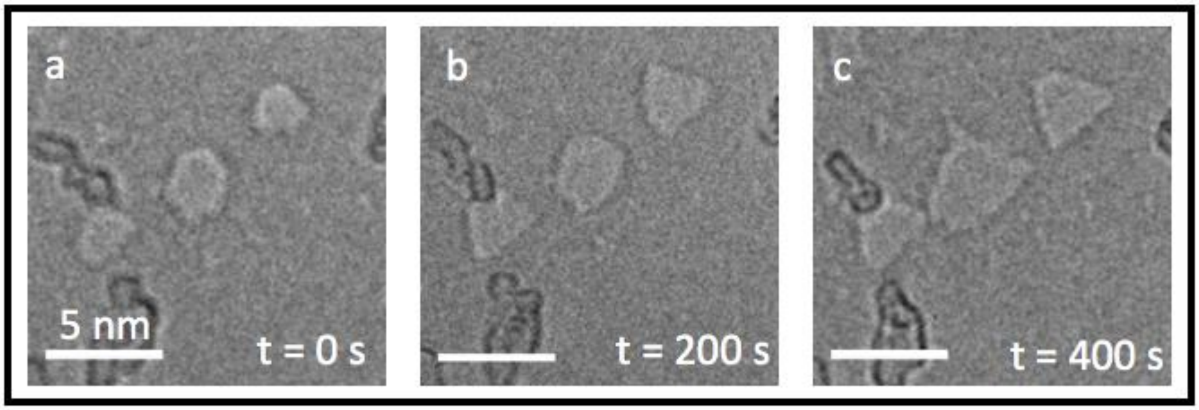

A time series of the evolution of hexagonal h-BN nanopores after the beam is reduced from ~70 A/cm^2 to ~20 A/cm^2 at time t = 0. Frames a.), b.), and c.) respectively show the sample after 0 seconds (image taken almost immediately upon spreading the beam), 200 seconds, and 400 seconds of exposure under the ~6 A/cm^2 beam. After the hexagonal shapes are formed at the the high beam current density, the vacancies grow into a triangle shape at the lower beam current density. All scale bars are 5 nm.
